# PanGEA: Identification of allele specific gene expression using the 454 technology

**DOI:** 10.1186/1471-2105-10-143

**Published:** 2009-05-14

**Authors:** Robert Kofler, Tatiana Teixeira Torres, Tamas Lelley, Christian Schlötterer

**Affiliations:** 1 University of Natural Resources and Applied Life Sciences, Department for Agrobiotechnology, Institute for Plant Production Biotechnology Konrad Lorenz Str 20, A-3430 Tulln, Austria; 2 Institut für Populationsgenetik, Veterinärmedizinische Universität Wien, Josef Baumann Gasse 1, 1210 Wien, Austria

## Abstract

**Background:**

Next generation sequencing technologies hold great potential for many biological questions. While mainly used for genomic sequencing, they are also very promising for gene expression profiling. Sequencing of cDNA does not only provide an estimate of the absolute expression level, it can also be used for the identification of allele specific gene expression.

**Results:**

We developed PanGEA, a tool which enables a fast and user-friendly analysis of allele specific gene expression using the 454 technology. PanGEA allows mapping of 454-ESTs to genes or whole genomes, displaying gene expression profiles, identification of SNPs and the quantification of allele specific gene expression. The intuitive GUI of PanGEA facilitates a flexible and interactive analysis of the data. PanGEA additionally implements a modification of the Smith-Waterman algorithm which deals with incorrect estimates of homopolymer length as occuring in the 454 technology

**Conclusion:**

To our knowledge, PanGEA is the first tool which facilitates the identification of allele specific gene expression. PanGEA is distributed under the Mozilla Public License and available at:

## Background

Next generation sequencing technologies hold great promise for biology in general [1]. They may be used to identify SNPs, pursue metagenomics, analyse DNA-protein interactions, and to discover non-coding RNA [2]. Furthermore, they may also be used for the analysis of the transcriptome [3,4] supplementing the microarray technology. Compared to microarrays, sequencing based analysis of the transcriptome allows to tackle new biological problems such as the identification of allele specific gene expression, absolute measurement of gene expression, identification of structural variation, identification of alternative splicing sites and cross species comparison of gene expression.

We developed PanGEA – The Comprehensive (ancient greek: pan) Gene Expression Analyzer – to enable a fast and user-friendly analysis of allele specific gene expression using the 454 technology. PanGEA can be used for quantification of gene expression, the identification of SNPs and the quantification of allele specific gene expression. Additionally, PanGEA implements a modification of the Smith-Waterman algorithm which deals with incorrect estimates of homopolymer length as occuring in the 454 technology.

PanGEA and the accompanying console applications have been mainly developed for Windows but also work in Linux and Mac OsX. PanGEA is distributed under the Mozilla Public Licence and can be obtained from  [see Additional file 1 for the executable and Additional file 2 for the source code of PanGEA].

## Implementation

### PanGEA-BlastN

To map ESTs to genes or whole genomes we developed PanGEA-BlastN. Similarly to Blast [5], PanGEA-BlastN uses an heuristic algorithm to find approximate hits between the database and the query sequence and then extends these hits with dynamic programming. PanGEA-BlastN is well-suited for mapping of EST reads obtained from next-generation sequencing technologies for the following reasons:

• the seeding (heuristic search for approximate hits) has been optimized. Pairwise alignments will only be created for the best seeds, which reduces the number of dynamic programming steps and thus computation time

• the necessity to map ESTs unambiguously is explicitly addressed

• the dynamic programming algorithm has been modified to deal with uncertainty of homopolymer length as occurring in the 454-technology or in the Helicos system [6,7]

• several modifications have been implemented which allow for introns in the EST sequences

The mapping algorithm of PanGEA-BlastN, initially builds a hash-table of the database sequence and subsequently scans for approximate hits between the query and the database sequence (seeds). Computation time is reduced by the identification of the best candidates for the highest scoring hit from the longest diagonals, i.e. longest succession of shared words between the query and the database sequence. Only the longest diagonals will be subjected to dynamic programming. In addition to the classic Smith-Waterman algorithm PanGEA-BlastN provides a modified Smith-Waterman algorithm which is especially adapted to uncertainty of homopolymer length estimates occurring in several next-generation sequencing technologies [6,7]. We also implemented improvements in the dynamic programming algorithm to increase computation efficiency Gotoh [8]. Unambiguously mapped ESTs are identified by comparing the scores of pairwise alignments. If the score difference between the best and the second best hit exceeds a minimum threshold, a mapping result is considered unambiguous. Ambiguous results are reported into a separate output file. PanGEA-BlastN also offers an intron-mode in which introns are already considered during seeding. Putative exons, separated by an intron, are individually aligned by dynamic programming (partial alignments) and subsequently aggregated into a composite alignment. Partial alignments, representing putative exons, are frequently overlapping with respect to the query sequence. For example, 'exon a' covering the bases 5 – 125 of a query sequence overlaps with 'exon b' which covers the bases 115 – 220. These overlaps are biologically not meaningful and have to be resolved. Therefore, PanGEA-BlastN calculates the alignment scores for each overlap individually and removes the overlap with the lowest score.

In contrast to other Blast-like approaches, insignificant hits cannot be filtered by specification of a minimum alignment score. Rather, spurious hits can be filtered after a PanGEA-BlastN search with the option 'Manage Pairwise alignments', by specifying a minimum similarity, alignment length and read coverage (see below). This has the advantage that performing a separate PanGEA-BlastN search for each different setting is not necessary. Instead, a PanGEA-BlastN search is conducted only once and the optimal parameters can subsequently be quickly estimated. The total length of the database sequences is only limited by the amount of available RAM, an analysis using the *D*. *melanogaster *genome as database sequence (120 Mbp) typically requires about 700 MB of RAM. No upper limit exists for the number of query sequences as PanGEA-BlastN operates in batch mode. PanGEA-BlastN is available as a stand-alone console application and embedded into a user-friendly GUI in the software PanGEA.

### Seeding

Identification of approximate hits between the database and the query sequence, i.e seeding, provides the starting point for subsequent dynamic programming steps. Since the most time consuming processes during mapping of ESTs is dynamic programming, minimizing the number of dynamic programming steps could considerably improve computational efficiency. For EST mapping to genes or genomes the primary interest is the identification of the corresponding genes, thus only a single best hit is expected for each EST. This particular requirement can be used to design an efficient EST-mapping-algorithm by searching, already during seeding, for best-hit-candidates and subsequently aligning only those with dynamic programming. In contrast to Blast which aligns each approximate hit between a database and a query sequence [5], PanGEA-BlastN only aligns the best-hit-candidates. Best-hit-candidates are identified by searching for the longest diagonals between a database and a query sequence [9,10]. Briefly, a hash table is built, containing each non-overlapping word of length *k *in the database sequences. Each word holds information about the index of the database sequence (*i*) and the position within the database sequence (*j*). Words having a low information content, i.e. occur several-fold more often than expected by chance (*n *> *n*_*max*_), are removed from the hash table. The maximum number of occurrences *n*_*max *_for words of length *k *in database sequences having the total length *l*_*d *_can be calculated as



**Figure 1:**
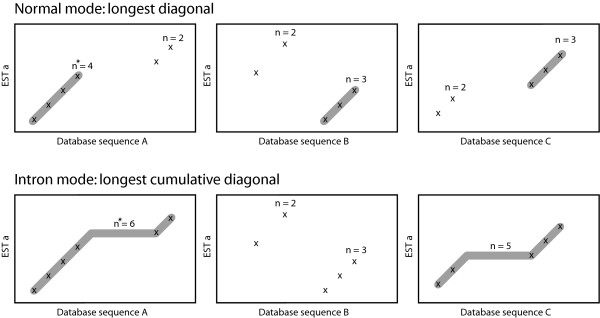
**Seeding during the two PanGEA-BlastN search modes**. Individual word positions are marked with an *x*. Length of each diagonal (*n*) is shown above whereas the longest diagonal is indicated by a star. Diagonals being passed as seeds to the dynamic programming algorithm are shown shaded (*n *≥ *n*_*longest *_- 1).

Where *c *denotes the low complexity cutoff specified by the user. After building a hash table, the query sequences are scanned. For each overlapping word of length *k *in the query sequence the corresponding matches in the hash table are identified. For these words, a shift (*s*) is calculated *s *= *j *- *t *where *j *is the position of the word in the database sequence and *t *the position in the query sequence. Subsequently, these words are sorted and parsed by searching for consecutive words with identical index (*i*) and identical (or similar) shift (*s*) [10]. A consecutive series of *n *identical indexes and shifts form a diagonal with length *n*. The algorithm searches for the longest diagonal, having the length *n*_*longest*_, and passes all diagonals with a length *n *≥ *n*_*longest *_- 1 as seeds to the dynamic programming algorithm (Fig. 1). The main difference to the algorithm of Ning et al. [10] is that PanGEA-BlastN uses the diagonals merely as seeds for dynamic programming. In addition to this, PanGEA-BlastN provides an optional modification to account for the presence of introns in the reads being mapped against genomic sequences. Consecutive diagonals of length *n *≥ 2 may be concatenated thus forming cumulative diagonals (Fig. 1). These cumulative diagonals allow for introns in the ESTs already during seeding. A maximum distance between the individual diagonals may be specified by the user.

### Homopolymer adapted dynamic programming

**Figure 2:**
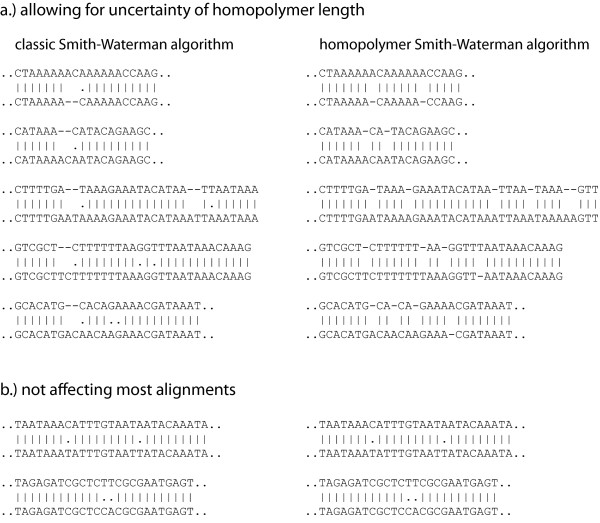
**Pairwise alignments created with the homopolymer Smith-Waterman algorithm compared to the classic Smith-Waterman algorithm [for the whole alignments see Additional file **3**]**.

Several next-generation sequencing technologies, for example the 454-platform or the Helicos system introduce new types of sequencing errors [11-13]. Most notably, the length of homopolymers is often estimated incorrectly [11-13,7]. These sequencing errors frequently cause the alignments of mismatching bases (Fig. 2), which can lead to wrong estimates of the evolutionary distance between two sequences or may complicate the identification of SNPs in downstream applications. We developed a novel Smith-Waterman algorithm, which accounts for this uncertainty of homopolymer length by allowing for gaps preferentially in homopolymers.

The basic idea of the algorithm is to adjust the gap-introduction penalty (gap-opening penalty) dynamically to the "homopolymer-terrain" of a nucleotide sequence, i.e to use a position specific gap-introduction penalty, which decreases linearly within hompolymers. Additionally, a reduced gap-introduction penalty should only be valid within the tract of a homopolymer, if a gap is to be extended beyond, the default gap-introduction penalty should be used.

Let the two DNA sequences be *D *= *d*_1_*d*_2_...*d*_*n *_(database) and *Q *= *q*_1_*q*_2_...*q*_*m *_(query). Let *I*_*max *_further be the default (maximum) gap-introduction penalty, *E *the gap-extension penalty, *S *the hit score and *P*_*mm *_the mismatch penalty, then the minimum gap-introduction penalty *I*_*min *_can be calculated.



Gap introduction penalties *I *<*I*_*min *_cause inconsistent alignments. Now two position specific gap introduction matrices can be constructed *I*_*D *_= *I*_*d*1_*I*_*d*2_...*I*_*dn *_and *I*_*Q *_= *I*_*q*1_*I*_*q*2_...*I*_*qm *_where each entry *I*_*di*_, *I*_*qk *_relates to an corresponding entry *d*_*i*_, *q*_*k *_in *D *and *Q *respectively, where 1 ≤ *i *≤ *n *and 1 ≤ *k *≤ *m*. The two matrices *I*_*D *_and *I*_*Q *_are instantiated with values for *I*_*di*_, *I*_*qk *_where *I*_*min *_= *I*_*di*_, *I*_*qk *_= *I*_*max*_. In the absence of homopolymers in sequences *D *and *Q*, the corresponding values *I*_*di *_and *I*_*qk *_respectively, are set to *I*_*max *_whereas these values decrease linearly to *I*_*min *_within homopolymers.

For gaps of length *t *the affine gap penalty *P*_*gt *_can be calculated [14]:



The homopolymer Smith-Waterman algorithm described here, additionally uses the homopolymer gap penalty *P*_*ht *_for gaps of length *t*.



To restrict the introduced low-penalty-gaps to homopolymers, we introduced the homopolymer-transgression-penalty *T*, where *x *denotes the number of homopolymer transgressions. A homopolymer is transgressed each time *q*_*i *_≠ *q*_*i*+1 _for insertions and *d*_*i *_≠ *d*_*i*+1 _for deletions. A high value of *T *restricts low-penalty-gaps exclusively to homopolymer tracts, whereas *T *= 0 allows an extension of these gaps without imposing any restrictions. Introduction of the homopolymer transgression penalty additionally has the advantage that this facilitates the implementation of the homopolymer Smith-Waterman algorithm in the important modification described by Gotoh [8].

Let *s*(*d*_*i*_, *q*_*k*_) be the similarity between the two bases *d*_*i *_and *q*_*k *_then a two dimensional matrix *H *can be constructed, similar as described by Smith and Waterman [14].



Fig 2 shows some pairwise alignments generated with the homopolymer Smith-Waterman algorithm compared to alignments generated by the classical Smith-Waterman implementation [for the whole alignments see Additional file 3].

We implemented this homopolymer Smith-Waterman algorithm together with the modification described by Gotoh [8], which reduces the required computation time from *O*(*m*^2^*n*) to *O*(*mn*) where m and n is the length of the database and the query sequence respectively [8]. We simply used four one-dimensional arrays which keep track of the highest possible gap score (normal gaps and homopolymer gaps, each in the database and the query sequence) instead of the two originally described. An implementation of this homopolymer Smith-Waterman algorithm is available as the stand-alone application 'PanGEA-SW'.

### Mapping statistics and management of pairwise alignments

The mapped cDNA sequence reads can be managed using the user-friendly GUI of PanGEA. Summary statistics for all ESTs mapping to the same gene are provided, such as the number of sense-ESTs mapping to the gene or the number of ESTs containing large gaps (putative introns). Subsets of the mapped reads can be displayed and exported by providing several quality criteria, such as ambiguity, minimum length of the alignment, minimum similarity, minimum coverage of the EST, presence or absence of large gaps (putative introns) or transcript orientation (sense, anti-sense). The subsets may be exported and used for a subsequent analysis, for example SNP identification.

### SNP identification

SNPs are identified from the pairwise alignments. If a list of validated SNPs is available, PanGEA provides the option to use only these SNPs for frequency estimates from the sequence reads. If no validated SNPs are available, PanGEA identifies SNPs from the sequence reads and provides several options to minimize the number of miscalled SNPs. PanGEA can account for the quality scores of the sequences, determining the sequence quality at the SNP-site and its neighborhood.

The strategy for SNP-identification in PanGEA is to first identify SNPs using not-stringent parameters and to subsequently select a subset of these SNPs with the option 'Manage SNPs' using stringent parameters. This has the main advantage that a separate SNP-identification for each different parameters is not necessary, rather SNPs are identified only once and subsets can be flexible selected. This approach allows for an interactive fine-tuning of the selected SNPs and SNP-alleles. To test the SNP identification module we created extensive unit tests using NUnit [see Additional file 4].

The SNP identification module is available as stand-alone console application 'PanGEA-SNP' and has been integrated into the software PanGEA.

### Identification of allele specific gene expression and visualisation of SNPs

**Figure 3:**
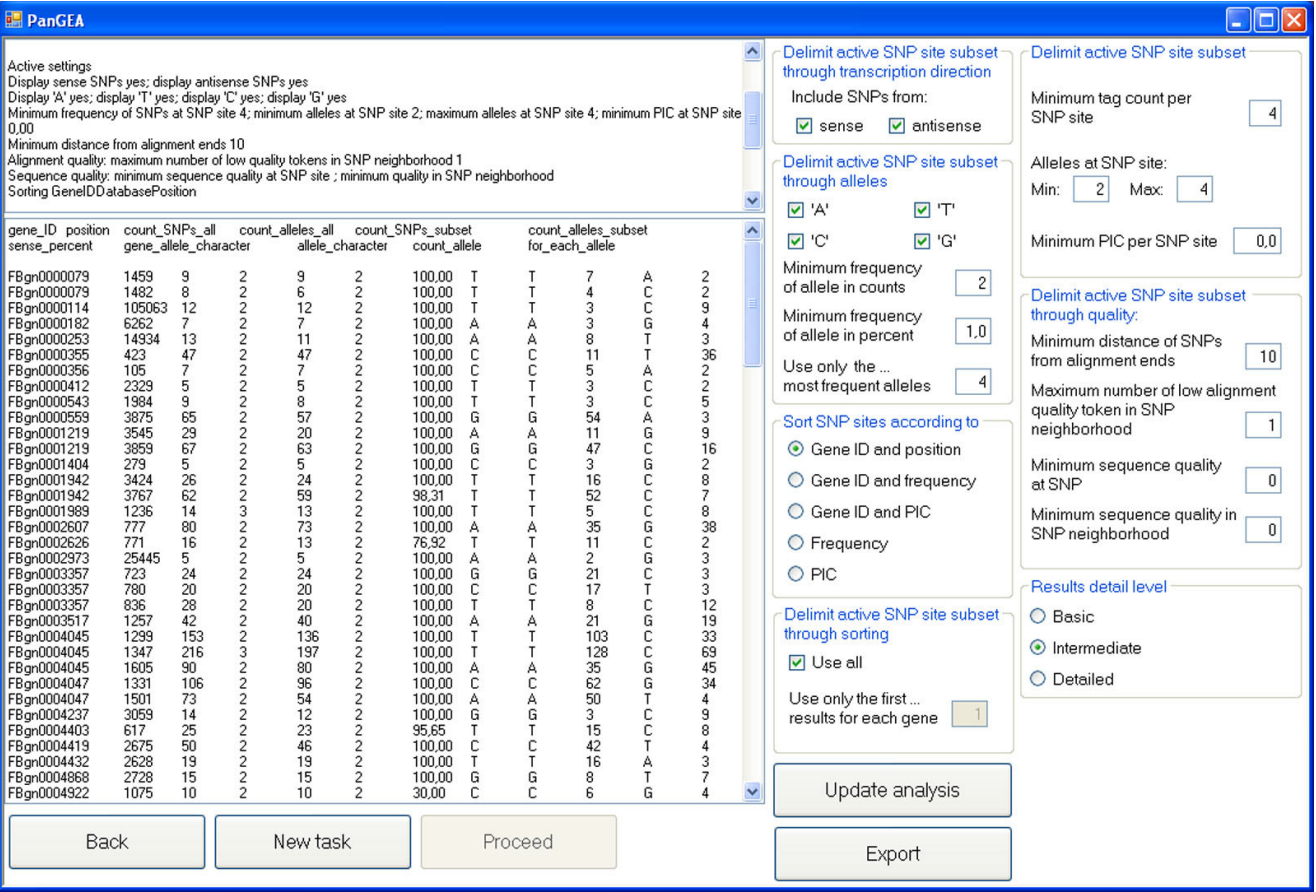
**Summary statistics for each SNP-site and quantification of allele specific gene expression by the frequencies of individual SNP-alleles**.

PanGEA provides two options to display the identified SNPs. Either summary results are displayed for each SNP-site (Fig. 3) or for each database sequence (typically corresponding to a gene or transcript). The summary statistics for each SNP-site furthermore provide a convenient way to quantify allele specific gene expression by displaying the SNP-allele frequencies at each SNP-site (Fig. 3). Optionally, subsets of the SNP-alleles can be displayed according to quality, direction of transcription (sense and anti-sense) and minimum frequency. The quality of SNP-alleles can be assessed by several criteria such as the minimum sequence quality of the SNP, the minimum sequence quality in the neighborhood of the SNP and the minimum distance from the alignment ends.

### Methods for benchmarking

We obtained the *Drosophila melanogaster *genome (release 5.5), gene sequences (release 5.5) and the transcripts (release 5.5) from Flybase . All benchmarks were carried out on a standard desktop computer with 2 GB of RAM and an Intel™Core Duo^®^2 × 2.4 GHz processor. For benchmarking, a set of 26 040 454-ESTs, with an average length of 106 bp, derived from the 3'-end of *D*. *melanogaster *transcripts, were downloaded from GenBank [accession numbers: EV574767 – EV600806; [4]]. These 454-ESTs were mapped to the genes of *D*. *melanogaster *using stand-alone BLAST 2.2.13 and PanGEA-BlastN. Both programs used only one of the two available processors. The following PanGEA-BlastN settings were used: word length 11; minimum diagonal 2; low complexity threshold 10; hit score 3; mismatch penalty 5; gap introduction penalty 11; gap extension penalty 2; homopolymer transgression penalty 3; ambiguity threshold 10; homopolymer Smith-Waterman; intron mode was off; The defaults settings were used for NCBI-BlastN, except the e-value was set to 10^-10 ^and the tabular output format (-m 8) was used. The pairwise alignments, resulting from the mapping of these 26 000 454-ESTs to the genes of *D*. *melanogaster*, were used for the subsequent identification of SNPs.

To test the performance of PanGEA-BlastN with the 454-platform in detail, we developed a console application which randomly excises 1000 ESTs from the transcripts of *D*. *melanogaster*, randomly introduces pseudo-sequencing-errors (0%, 5% and 10%) into these ESTs and maps them either to the genes or the whole genome of *D*. *melanogaster *using PanGEA-BlastN. An EST was considered correctly mapped to the genes, if the gene-ID (specified in header of transcript) matched the mapping result, whereas an EST was considered correctly mapped to the whole genome, if the chromosome-ID as well as the position within the chromosome (specified in header of transcript) matched the mapping result.

## Results

### Influence of the mapping parameters used by PanGEA-BlastN

We evaluated the influence of the PanGEA-BlastN parameters on the mapping accuracy and computation time by mapping 1000 randomly generated 250 bp fragments from *D. melanogaster *transcripts (release 5.5) to the corresponding genes.

**Figure 4:**
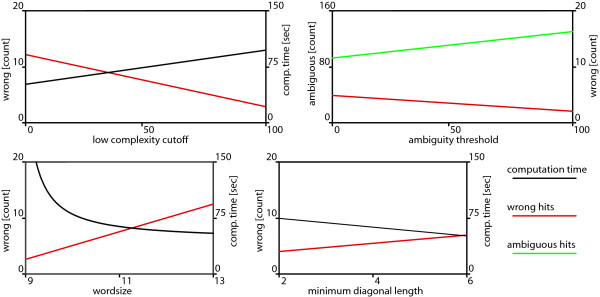
**Effect of the most important parameters on the performance of PanGEA-BlastN**. Values were calculated for mapping of 1000 randomly generated 250 bp fragments from *D*. *melanogaster *transcripts to the corresponding genes. Benchmarks were calculated in triplicate for five (or more) datapoints [see Additional file 5]. If not stated otherwise the following parameters were used: word length 11; minimum diagonal 3; low complexity threshold 20; ambiguity 12.

First, we determined the influence of the low complexity cutoff (c), which reflects the maximum frequency of a word in a hash-table. Words occurring c times more frequent than expected by chance were not considered. As expected the mapping accuracy increased with 'c' on the expense of computation time (Fig. 4a). Nevertheless, the number of inaccurately mapped 454-ESTs was low (< 1.20%) irrespective of the low complexity cutoff.

Next we calculated the influence of the ambiguity threshold, which measures the difference between the best and the second best hit. Increasing the ambiguity threshold resulted in a moderate reduction for incorrectly mapped 454-ESTs. While < 1.0% were mapped incorrectly when only the best hit (ambiguity threshold = 0) was considered, an ambiguity threshold of 100 had < 0.5% incorrectly mapped 454-ESTs. The trade off of this increase in mapping accuracy was an increase of ambiguously mapped 454-ESTs. Rather than 9% for the best hit, an ambiguity threshold of 100 resulted in 13% ambiguous hits (Fig. 4b). The ambiguity threshold only has a minor influence on computation time [see Additional file 5]. On the other hand, increasing the word size dramatically reduces the computation time on the expense of the mapping accuracy (Fig. 4c). The last parameter evaluated was the 'minimum diagonal length'. Similar to word size an increase in minimum diagonal length reduced the computational time on the expense of mapping accuracy (Fig. 4c).

These results illustrate that optimal parameters represent a compromise between computation time, specificity and sensitivity.

### Mapping performance of PanGEA-BlastN

To assess the performance PanGEA-BlastN we compared PanGEA-BlastN with NCBI-BlastN. A set of more than 25,000 454 ESTs [4], with an average length of 106 bp were mapped to their gene sequences using PanGEA-BlastN and NCBI-BlastN. Despite a considerable reduced computation time, PanGEA-BlastN generated very similar results as NCBI-BlastN (Table 1), suggesting that the simplified search did not compromise the mapping efficiency.

**Table 1:** Comparision of the performance of PanGEA-BlastN with NCBI-BlastN [5].

	NCBI	PanGEA	*P *∩ *N*^1^
Time	47 min	6 min	-
Hits	23 512	24 600	23 436
Ambiguous	1 787	1 887	1 615

More than 25,000 454-ESTs from *D*. *melanogaster *[4] were mapped to their genes. Ambiguity was reported if the score of the best hit differed from the score of the second best hit by less than 10.

^1^*PanGEA *∩ *NCBI*, i.e mapping results in which both tools agree

Nevertheless, we noted some differences between PanGEA-BlastN and NCBI-BlastN. To evaluate the mapping efficiency of PanGEA-BlastN, we computationally generated 1000 454-EST-like sequences from *D. melanogaster *transcripts and mapped them either to gene sequences (including intronic sequences) or to the entire genome. To account for sequencing errors, we also introduced 5% and 10% mutations prior to mapping.

The performance of PanGEA-BlastN was assessed using the following criteria: (i) the number of ambiguous hits (ii) the number of correct hits, including ambiguous hits containing the correct hit, (iii) the number of wrong hits, including ambiguous hits not containing the correct hit (iv) the number of not-mapped ESTs, (v) the number of identified large gaps (> 50 bp; putative introns) and finally (vi) the required computation time.

A very high proportion (> 99.5%) of the ESTs was correctly mapped irrespectively of the sequence divergence (Table 2). This mapping accuracy could be further improved by changing some of the parameters, such as word size (see previous section). We noted a substantial discrepancy of unambiguously mapped reads for the gene sequences and genomic sequences. Despite a higher complexity, fewer reads (2.5%) were ambiguously mapped to the genome than to the gene sequences (10%). The reason for this discrepancy are ovelapping/nested genes (data not shown). Most importantly, the mapping accuracy was not effected when the intron discovery mode was switched on. However, several large gaps (i.e.: introns) were discovered, emphasizing the need for the intron discovery mode. Increasing the length of the 454-ESTs beyond 100 bp did not result in an increased mapping efficiency, suggesting that this length is sufficient for reliable mapping.

**Table 2:** Performance of PanGEA-BlastN with the 454-platform using the recommended settings.

tag-to-gene mapping^1^													
		normal mode						Intron mode					
L^2^	s^3^	a^4^	c^5^	w^6^	n^7^	i^8^	t^9^	a^4^	c^5^	w^6^	n^7^	i^8^	t^9^
	100	126	997	1	2	6	10	111	993	5	2	63	10
100	95	95	932	6	62	0	11	104	952	6	42	11	11
	90	42	408	13	579	0	4	35	450	10	540	0	5
	100	86	993	5	2	87	34	108	991	8	1	206	40
200	95	88	988	8	4	88	38	91	994	5	1	170	41
	90	90	953	5	42	44	37	78	885	13	102	75	36
	100	114	986	10	4	211	84	94	996	3	1	407	90
300	95	86	988	10	2	188	81	102	990	6	4	354	95
	90	103	984	8	8	162	85	99	992	7	1	263	93
	100	74	994	4	2	312	128	85	988	7	5	574	153
400	95	87	986	12	2	300	137	79	981	13	6	499	153
	90	78	986	14	0	250	150	85	993	4	3	366	151

													
tag-to-genome mapping^10^													

	100	32	1000	0	0	11	11	21	998	1	1	42	10
100	95	27	973	2	25	1	14	23	984	2	14	24	22
	90	14	399	4	597	0	14	11	337	13	650	1	4
	100	31	997	3	0	93	59	25	994	6	0	190	55
200	95	31	993	7	0	82	49	20	995	5	0	154	51
	90	20	961	1	38	42	45	12	956	1	43	99	47
	100	26	998	2	0	214	94	27	997	3	0	341	107
300	95	16	998	2	0	178	96	21	995	5	0	285	113
	90	23	972	9	19	151	99	21	989	10	1	250	102
	100	21	999	1	0	328	194	20	998	2	0	496	181
400	95	15	998	2	0	287	144	23	993	7	0	422	178
	90	19	996	4	0	260	168	20	992	8	0	400	168

Values were calculated for mapping 1000 randomly excised ESTs, either to the genes or to the whole genome of *D. melanogaster*

^1 ^settings: word length 11; minimum diagonal 3; low complexity threshold 10; homopolymer Smith-Waterman algorithm; no maximum intron length

^2 ^length of the tags in bp

^3 ^similarity of the tag with the target sequence in percent

^4 ^ambiguous mapping results; min score difference for unambiguous best hit 12

^5 ^correctly mapped tags (including ambiguous results containing the correct hit)

^6 ^wrongly mapped tags (including ambiguous results not containing the correct hit)

^7 ^no hit identified

^8 ^number of long gaps (> 50 bp), putative introns

^9 ^mapping time in seconds, without the time required for constructing the word hash-table

^10 ^settings as above, only the maximum intron length was set to 5000 bp

However, considering the benchmarks of Table 2 we recommend the following settings for mapping of 454-ESTs, which are an attempt to optimize the antagonistic demands for efficiency, sensitivity and specificity: word length 11 (10–12), minimum diagonal length 3 (2–3), low complexity cutoff 10 (10–50); intron mode on. These settings are used as defaults by PanGEA-BlastN.

## Discussion and conclusion

PanGEA provides an important step towards the use of massively parallel sequencing for gene expression analysis. While it is currently not apparent which of the new sequencing technologies will provide the most appropriate tool for gene expression analysis, the software tool PanGEA allows an accurate quantification of allele specific gene expression.

## Availability and requirements

Project name: PanGEA

Project home page: 

Operating system(s): Windows, Linux and Mac Os X

Programming language: C#

Other requirements: .Net Framework 2.0 for Windows; Mono 2.0 for Mac Os X and Linux

License: Mozilla Public License

Any restrictions to use by non-academics: none

## Authors' contributions

RK, TTT and CS conceived the project. RK did the programming. CS supervised the project. RK, TTT, TL and CS wrote the manuscript. All authors read and approved the final manuscript.

## Supplementary Material

Additional file 1 **PanGEA 1.04**. A platform independent executable of PanGEA. Click here for file

Additional file 2 **PanGEA source code**. The source code of PanGEA. Click here for file

Additional file 3 **Comparison of alignments**. Compares the pairwise alignments created with the homopolymer Smith-Waterman algorithm to the classic Smith-Waterman algorithm. Click here for file

Additional file 4 **NUnit test for the SNP identification module**. A zip file containing the unit text for the SNP modification mudule as C# code. Click here for file

Additional file 5 **Performance of PanGEA-BlastN**. Provides detailed benchmarks for PanGEA-BlastN. Click here for file
